# Developing and validating Parkinson’s disease subtypes and their motor and cognitive progression

**DOI:** 10.1136/jnnp-2018-318337

**Published:** 2018-07-25

**Authors:** Michael Lawton, Yoav Ben-Shlomo, Margaret T May, Fahd Baig, Thomas R Barber, Johannes C Klein, Diane M A Swallow, Naveed Malek, Katherine A Grosset, Nin Bajaj, Roger A Barker, Nigel Williams, David J Burn, Thomas Foltynie, Huw R Morris, Nicholas W Wood, Donald G Grosset, Michele T M Hu

**Affiliations:** 1 Department of Population Health Sciences, University of Bristol, Bristol, UK; 2 Nuffield Department of Clinical Neurosciences, Division of Clinical Neurology, University of Oxford, Oxford, UK; 3 Oxford Parkinson’s Disease Centre, University of Oxford, Oxford, UK; 4 Institute of Applied Health Sciences, University of Aberdeen, Aberdeen, UK; 5 Department of Neurology, Institute of Neurological Sciences, Glasgow, UK; 6 Department of Neurology, Queen’s Medical Centre, Nottingham, UK; 7 Clinical Neurosciences, John van Geest Centre for Brain Repair, Cambridge, UK; 8 Cardiff University, Institute of Psychological Medicine and Clinical Neurosciences, Cardiff, UK; 9 Faculty of Medical Sciences, Newcastle University, Newcastle, UK; 10 Sobell Department of Motor Neuroscience, UCL Institute of Neurology, London, UK; 11 Department of Clinical Neuroscience, UCL Institute of Neurology, London, UK; 12 Department of Molecular Neuroscience, UCL Institute of Neurology, London, UK

## Abstract

**Objectives:**

To use a data-driven approach to determine the existence and natural history of subtypes of Parkinson’s disease (PD) using two large independent cohorts of patients newly diagnosed with this condition.

**Methods:**

1601 and 944 patients with idiopathic PD, from Tracking Parkinson’s and Discovery cohorts, respectively, were evaluated in motor, cognitive and non-motor domains at the baseline assessment. Patients were recently diagnosed at entry (within 3.5 years of diagnosis) and were followed up every 18 months. We used a factor analysis followed by a k-means cluster analysis, while prognosis was measured using random slope and intercept models.

**Results:**

We identified four clusters: (1) *fast motor progression* with symmetrical motor disease, poor olfaction, cognition and postural hypotension; (2) *mild motor and non-motor disease* with intermediate motor progression; (3) *severe motor disease, poor psychological well-being* and *poor sleep* with an intermediate motor progression; (4) *slow motor progression* with tremor-dominant, unilateral disease. Clusters were moderately to substantially stable across the two cohorts (kappa 0.58). Cluster 1 had the fastest motor progression in Tracking Parkinson’s at 3.2 (95% CI 2.8 to 3.6) UPDRS III points per year while cluster 4 had the slowest at 0.6 (0.1–1.1). In Tracking Parkinson’s, cluster 2 had the largest response to levodopa 36.3% and cluster 4 the lowest 28.8%.

**Conclusions:**

We have found four novel clusters that replicated well across two independent early PD cohorts and were associated with levodopa response and motor progression rates. This has potential implications for better understanding disease pathophysiology and the relevance of patient stratification in future clinical trials.

## Introduction

Parkinson’s disease (PD) is a progressive neurodegenerative disorder characterised by a wide range of motor and non-motor features, for which there is no known cure. However, therapeutic strategies might soon be available with prolonged benefits that could affect the underlying pathogenesis, and hence delay or ultimately prevent the inexorable course of this disease. To date, none of the 16 drugs evaluated for PD disease modification have succeeded in phase III trials, with a further eight compounds currently in the discovery pipeline.^1^ PD is an inherently complex disorder with known heterogeneity in terms of clinical presentation as well as rate of progression and risk of disease complications. The basis for this is only now starting to be understood, in terms of the role of genetic factors, for example, glucocerebrosidase gene mutations. The implications for future clinical trial design—if patient heterogeneity is ignored at baseline study selection, leading to potential confounds and misinterpretation of subsequent progression/complication rates—are highly significant.

Few naturalistic cohort studies in PD have been undertaken using large numbers of representative, community-ascertained patients, unselected on the basis of age or family history, and prospectively followed early from diagnosis. Such cohorts would more faithfully recapitulate disease evolution in the true-to-life populations encountered in clinical practice, where disease progression reflects both pathophysiology and any treatment effects, as reported in the CamPaIGN study.^2^

Data-driven approaches to delineate subtypes using cohorts of incident PD as well as cross-sectional studies^3–7^ have hypothesised that there are different PD subtypes. Better defining these subtypes will be important for understanding the aetiology of the disease, discovering biomarkers related to prognosis and for stratified medicine, including the discovery and response to new medications.^8^ In this study, we sought to better explore this aspect of PD using two large independent cohorts of newly diagnosed PD and in particular the number of distinct disease subtypes, their levodopa responsiveness and rate of motor and cognitive decline. This extends our previous work in this area using only one of the two cohorts (Discovery), without assessing levodopa responsiveness or the subsequent rate of motor and cognitive decline.^9^

## Materials and methods

### Patient populations

Tracking Parkinson’s is a prospective cohort of recently diagnosed patients with PD who were recruited from around the UK between February 2012 and May 2014. Full details of this cohort and full inclusion/exclusion criteria have been published elsewhere.^10^ The Oxford Parkinson’s Disease Centre Discovery cohort (hereafter referred to as Discovery) is also a prospective cohort of recently diagnosed patients with PD who were recruited from 11 hospitals in the Thames Valley region between September 2010 and January 2016. Full details of the Discovery cohort and full inclusion/exclusion criteria have also been published elsewhere.^11^ In both studies, patients were defined as recently diagnosed if recruited within 3.5 years of diagnosis. In order to exclude patients with similar conditions that may have been incorrectly diagnosed as PD, we only included individuals in both cohorts if they had a probability of PD ≥90% as rated by a research neurologist/movement disorder specialist at their latest visit. Patients have been (and are continuing to be) followed up every 18 months. Both studies were funded by Parkinson’s UK.

### Patient evaluation

Assessments of patients were via self-completed questionnaires and from outpatient clinics using standardised and validated scales both at baseline and follow-up. Variables used in this analysis were those adopted in our original cluster analysis paper^9^ and which were also included in the Tracking Parkinson’s cohort, and these included the Movement Disorders Society (MDS) revised Unified Parkinson’s Disease Rating Scale (UPDRS), where part III was measured in the ‘on’ state; Big Five Inventory; Epworth Sleepiness Scale; REM Sleep Behaviour Disorder Screening Questionnaire; Hospital Anxiety and Depression Scale; Questionnaire for Impulsive-Compulsive Disorders in Parkinson’s Disease; Honolulu Asia Aging Study Constipation Questionnaire; Montreal Cognitive Assessment (MoCA) adjusted for education years; Semantic verbal fluency (animals); Orthostatic blood pressure measurement; and Sniffin’ 16 odour identification scores. The levodopa equivalent daily dose (LEDD) was calculated from medication use questionnaires using established formulae.^12^ In addition, a levodopa challenge was carried out giving us a quantitative measure of response to medication (see online supplementary e-appendix for more details on methods).

10.1136/jnnp-2018-318337.supp1 Supplementary data

### Statistical analysis

We imputed missing data using the mean score if 80% or more questions were answered in any given test. Additionally, missing baseline data were imputed using the chained equations approach separately in the two cohorts. Factor analysis was used as a variable reduction technique on all the baseline phenotypic variables (details in online supplementary e-appendix). We then derived the clusters by using a k-means analysis of the factor scores and other baseline phenotypic variables not loading into one of the factors. Variables were standardised separately within each cohort to ensure that each variable had the same weighting within the k-means analysis. Further details are described in our previous publication.^9^

A discriminant analysis model was then fitted to the Tracking Parkinson’s clusters and used to predict clusters within the Discovery cohort. These predicted clusters were compared with the k-means clusters in the Discovery cohort to determine the stability of our approach. We used the kappa statistic to compute the extent of agreement and adopted accepted guidelines^13^ to determine the strength of this agreement.

We then carried out a between-cluster comparison of a range of clinical and demographic variables, which had not been used in the estimation of the clusters using analysis of variance and χ^2^ tests. We modelled important disease-related variables (UPDRS III and MoCA scores) longitudinally using multilevel random slope and intercept models to estimate disease progression by cluster. A sensitivity analysis using pattern-mixture models was carried out to determine whether patients lost to follow-up may potentially have biased our disease progression estimates.^14^

## Results

We analysed data on 1601 patients in Tracking Parkinson’s and 944 in Discovery (online web supplementary figure 1). Both cohorts had around 35% women, were predominantly white (>98%) and had an average age of diagnosis of about 66 years (see table 1). The disease duration from diagnosis was on average 1.2–1.3 years. Compared with Tracking Parkinson’s, the Discovery cohort had more severe motor disease as measured by the UPDRS III and disease severity as measured by the Hoehn and Yahr or the sum score of UPDRS parts I–IV (p<0.001), and slightly worse average cognition as measured by the MoCA. However, the Tracking Parkinson’s cohort had worse motor aspects of experiences of daily living (UPDRS II) and motor complications (UPDRS IV) and had fewer untreated patients.

10.1136/jnnp-2018-318337.supp2 Supplementary data

**Table 1:** Demographic and clinical characteristics at baseline for the patients in the two studies

Variable	Tracking Parkinson’s cohort (n=1601) mean (SD) or n (%)	Discovery cohort (n=944) mean (SD) or n (%)	P-value difference between cohorts
Female	554 (34.6%)	334 (35.4%)	0.69*
Ethnicity (non-white)	28 (1.8%)	20 (2.1%)	0.51*
Age diagnosis (years)	65.9 (9.3)	65.9 (9.6)	0.92†
Disease duration from diagnosis (years)	1.3 (0.9)	1.2 (0.9)	0.03†
MDS-UPDRS part I‡	9.1 (5.2)	8.8 (5.1)	0.09†
MDS-UPDRS part II‡	9.5 (6.2)	8.7 (6.0)	<0.001†
MDS-UPDRS part III‡	22.3 (11.9)	26.4 (10.8)	<0.001†
MDS-UPDRS part IV‡	0.7 (1.7)	0.3 (1.1)	<0.001†
MDS-UPDRS total (all four parts)‡	41.8 (18.7)	44.2 (17.5)	0.002†
MoCA (adjusted for education years)‡	25.4 (3.4)	25.0 (3.3)	0.012†
Untreated	149 (9.3%)	119 (12.6%)	0.01*
LEDD (mg)	293 (205)	282 (212)	0.20†
LEDD (those on medication) (mg)	324 (190)	327 (194)	0.77†
Hoehn and Yahr§ median (IQR)	1 (1–2)	2 (2–2)	<0.001*

Motor assessments (UPDRS and Hoehn and Yahr) were rated in the clinically defined ‘on medication’ state for treated patients with PD.

*χ^2^ test.

†T-test.

‡Changed denominator where 80% or more of questions were answered.

§In Tracking Parkinson’s cohort, Hoehn and Yahr 1.5 and 2.5 were changed to 1 and 2, respectively, for comparison with Oxford Parkinson’s Disease Centre Discovery cohort.

LEDD, levodopa equivalent daily dose; MDS, Movement Disorders Society; MoCA, Montreal Cognitive Assessment; UPDRS, Unified Parkinson’s Disease Rating Scale.

**Figure 1:**
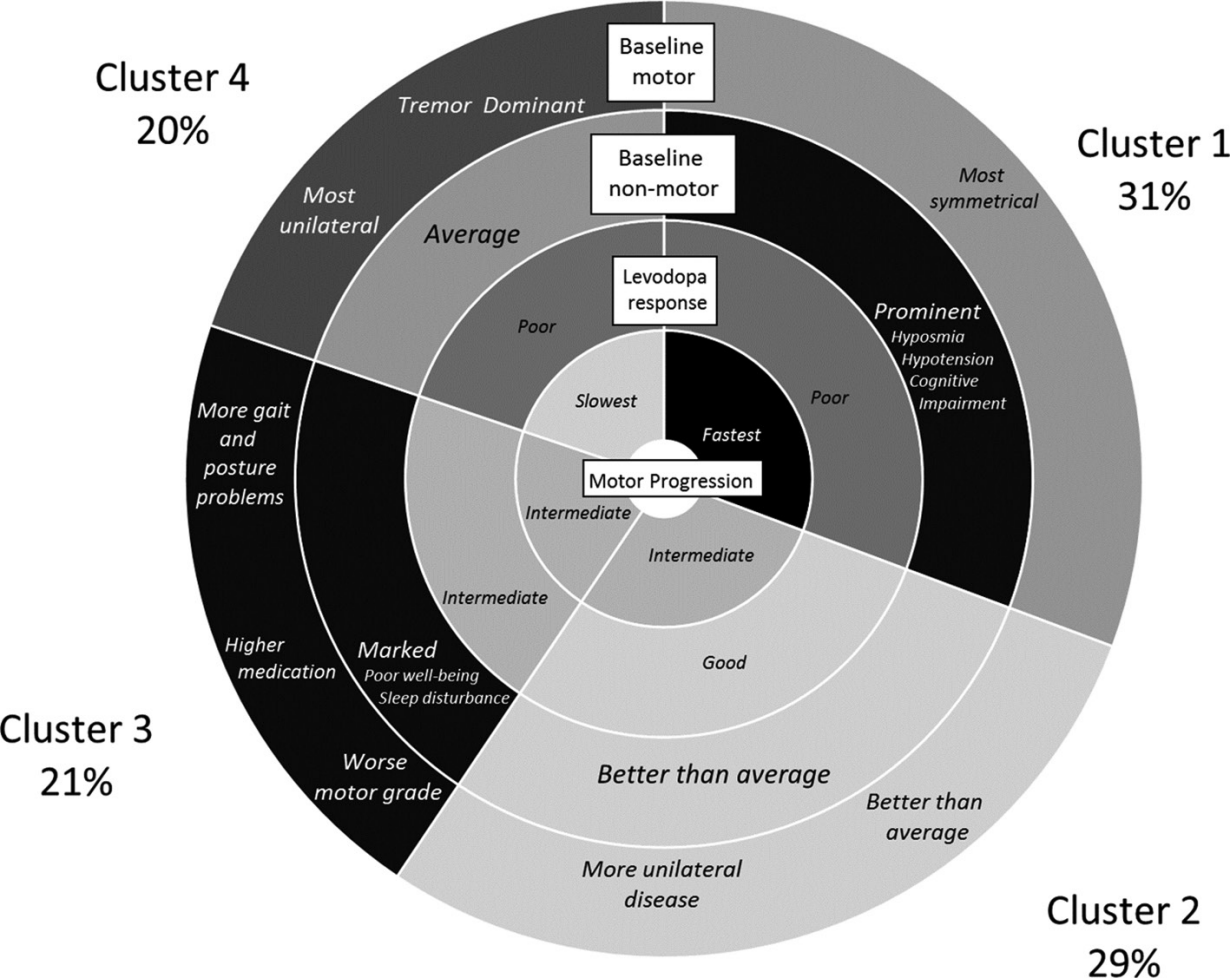
Important salient clinical features of the four clusters across the two cohorts where the percentages within each cluster are from the Tracking Parkinson’s cohort.

### Cluster analysis

**Figure 2:**
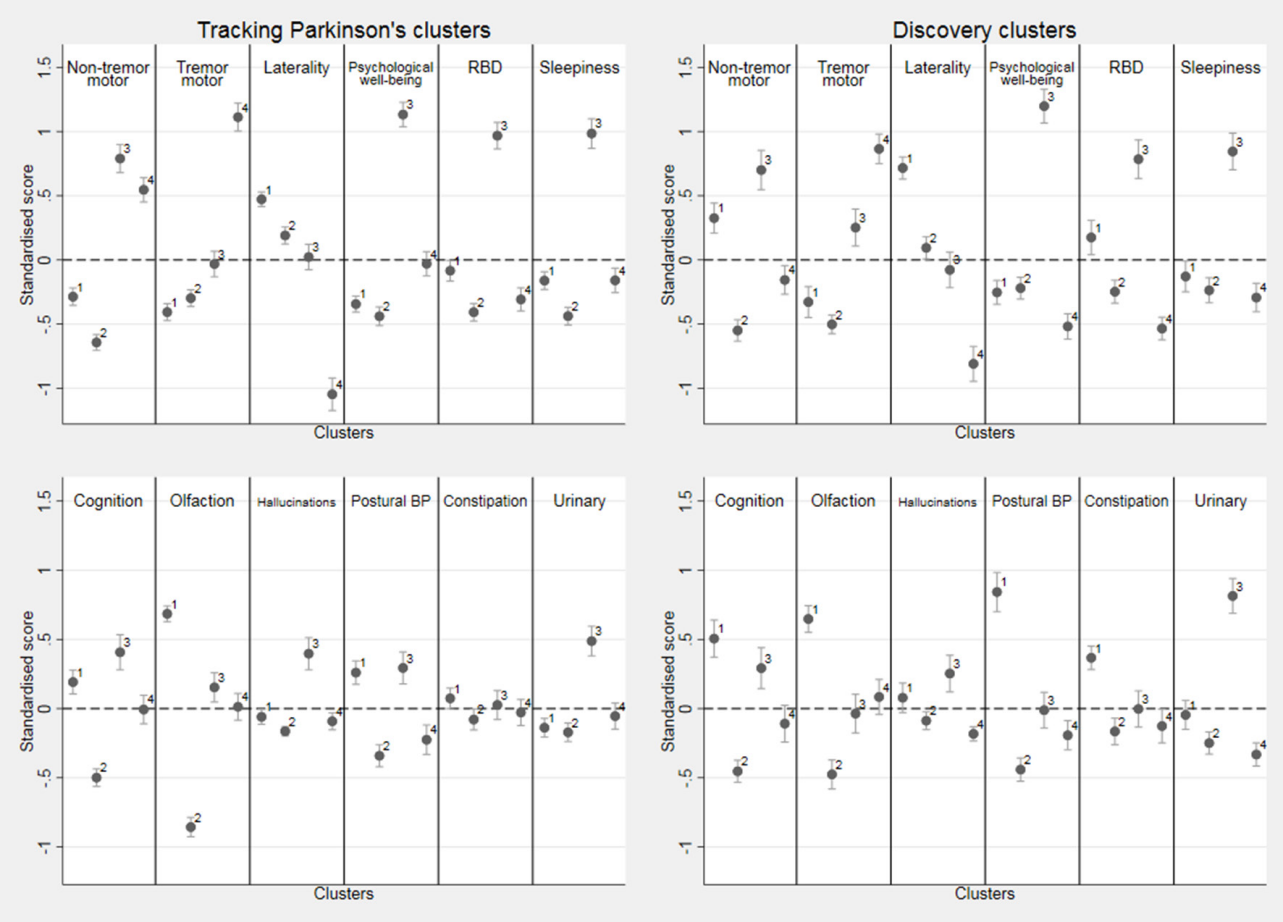
Within cluster means of the standardised variables for the four k-means cluster solution in both cohorts along with the 95% CI for the mean. Positive (above the dotted line) is worse than average and negative better than average. For laterality, positive is more bilateral than average and negative more unilateral than average. Note that hallucinations, constipation and urinary are categorical variables and were standardised using a slightly different method (see online supplementary appendix 1). In Tracking Parkinson’s, cluster 1 n=493 patients, cluster 2 n=459, cluster 3 n=336 and cluster 4 n=313, while in Discovery, cluster 1 n=218, cluster 2 n=319, cluster 3 n=196 and cluster 4 n=211. BP, blood pressure; RBD, rapid eye movement sleep behaviour disorder.

In our factor analysis, we found two factors: a psychological well-being and a non-tremor motor factor (table 2), as we reported previously.^9^ This shows that within our baseline phenotypic variables, we had multiple variables related to psychological well-being and to non-tremor motor function that were highly correlated. Using the statistics in web supplementary table 1 helped us decide that four clusters gave us an optimal solution. Figure 1 highlights the important features of the clusters and figure 2 shows the average of each of the standardised variables within each cluster for the Tracking Parkinson’s and Discovery cohorts. The groups were arbitrarily ordered in terms of size for Tracking Parkinson’s, but for the Discovery cohort they were ordered by similarity to the Tracking Parkinson’s clusters. In general, the cluster patterns between the cohorts were fairly similar but with some differences (see below). Details of the clusters are available in web supplementary table 2, which shows all the scores from the different tests included in the cluster analysis and categorised scores using standard cut-points from the literature for easier clinical interpretation. More details of the factor and the cluster analysis can be found in the online supplementary e-appendix.

**Table 2:** Confirmatory factor analysis within the Tracking Parkinson’s cohort showing standardised factor loadings of variables selected from exploratory factor analysis and measures of model fit

Variable	Factor 1 Psychological well-being	Factor 2 Non-tremor motor
MDS-UPDRS I apathy	0.512	
MDS-UPDRS I fatigue	0.599	
MDS-UPDRS I pain	0.544	
BFI—neuroticism	0.459	
HADS anxiety	0.795	
HADS depression	0.863	
QUIP	0.307	
MDS-UPDRS III speech		0.420
MDS-UPDRS III rigidity subscore		0.535
MDS-UPDRS III bradykinesia subscore		0.769
MDS-UPDRS III postural subscore		0.609
CFI=0.909		
TLI=0.932		
RMSEA=0.063		

CFI, TLI and RMSEA are all measures of model fit.

BFI, Big five inventory; QUIP, Questionnaire for Impulsive-Compulsive Disorders in Parkinson's disease.

CFI, Comparative Fit Index; HADS, Hospital Anxiety and Depression Scale; MDS, Movement Disorders Society; RMSEA, root mean square error of approximation; TLI, Tucker-Lewis Index; UPDRS, Unified Parkinson’s Disease Rating Scale.

The following describes the clusters observed in Tracking Parkinson’s (unless otherwise stated). The *fast motor progression* (1) cluster had less advanced motor features and psychological well-being but worse than average non-motor features such as blood pressure postural drop, olfaction and cognition with more symmetrical motor disease. However, within the Discovery cohort, the non-tremor motor was worse, rather than better than average, for this cluster. The *mild motor and non-motor disease* (2) cluster showed a milder form of the disease being better in most domains and was similar in the Discovery cohort analysis. The *severe motor disease, poor psychological well-being* and *poor sleep* (3) cluster was similar in the two cohorts and showed a more severe form of PD, especially in non-tremor motor features particularly bradykinesia and postural scores, worse psychological well-being and poor sleep and excessive daytime somnolence. The *slow motor progression* (4) cluster had severe tremor with unilateral disease and was similar in Discovery except for the fact that the non-tremor motor features were better than average in Discovery and worse than average in Tracking Parkinson’s.

Web supplementary table 3 shows the agreement between the k-means clusters in Discovery and those predicted by the Tracking Parkinson’s discriminant model. This reveals an overall agreement of 67.9% and a kappa value of 0.58 (95% CI 0.54 to 0.61) indicating moderate to substantial agreement.^13^ The major inconsistency comes in the *mild motor and non-motor disease* (2) cluster where 110 (34.5%) individuals are wrongly predicted to be in the *fast motor progression* (1) cluster.

### Clinical and demographic correlates of the clusters

The focus for the rest of this paper is on the clusters predicted from the larger Tracking Parkinson’s model and applied to the Discovery cohort because future patients would be classified from their baseline measurements into predicted clusters. We found a modest difference in disease duration since diagnosis (maximum average difference 3.5 months) between the clusters in both cohorts (table 3) but did not regard this as being clinically important in terms of explaining differences in phenotype. There was evidence of differences in gender, age at diagnosis, motor phenotype, Hoehn and Yahr stage, and medication use at baseline across the four clusters in both cohorts (p<0.001 in all variables) (see table 3). The *mild motor and non-motor disease* (2) cluster had the highest proportion of women and youngest age at diagnosis, while the *fast motor progression*(1) cluster had the highest age at diagnosis. The *severe motor disease, poor psychological well-being*and *poor sleep* (3) cluster had the highest proportion with the postural instability gait difficulty (PIGD) phenotype while the *slow motor progression* (4) cluster had the highest proportion of tremor-dominant disease at baseline. The LEDD at baseline was highest in the *severe motor disease, poor psychological well-being* and *poor sleep*(3) cluster, which also had the smallest proportion of untreated patients.

**Table 3:** Association of clusters with variables not used in the cluster analysis, along with a p value derived from a hypothesis test that the variable is equally distributed (ie, same mean or same proportion) among the four clusters

Variable	Tracking Parkinson’s clusters						Discovery—clusters predicted from Tracking Parkinson’s model					
	P values	Total (N=1601)	Cluster 1 (N=493, 30.8%)	Cluster 2 (N=459, 28.7%)	Cluster 3 (N=336, 21.0%)	Cluster 4 (N=313, 19.6%)	P values	Total (N=944)	Cluster 1 (N=307, 32.5%)	Cluster 2 (N=167, 17.7%)	Cluster 3 (N=223, 23.6%)	Cluster 4 (N=247, 26.2%)
Women*	<0.001	554 (34.6%)	144 (29.2%)	204 (44.4%)	98 (29.2%)	108 (34.5%)	<0.001	334 (35.4%)	92 (30.0%)	87 (52.1%)	58 (26.0%)	97 (39.3%)
Disease duration from diagnosis†	<0.001	1.3 (0.9)	1.3 (0.9)	1.2 (0.9)	1.5 (0.9)	1.4 (0.9)	0.002	1.2 (0.9)	1.2 (0.9)	1.1 (0.9)	1.4 (0.9)	1.2 (0.9)
Age diagnosis†	<0.001	65.9 (9.3)	68.1 (8.1)	62.6 (9.3)	66.5 (9.8)	66.7 (9.2)	<0.001	65.9 (9.6)	67.6 (8.8)	62.7 (9.4)	67.0 (9.5)	65.1 (10.2)
Age diagnosis <50*	<0.001	98 (6.1%)	16 (3.2%)	51 (11.1%)	19 (5.7%)	12 (3.8%)	<0.001	60 (6.4%)	8 (2.6%)	18 (10.8%)	12 (5.4%)	22 (8.9%)
UPDRS motor phenotype*‡												
Tremor dominant	<0.001	741 (48.0%)	194 (40.8%)	241 (54.9%)	92 (28.3%)	214 (70.6%)	<0.001	510 (54.7%)	129 (43.0%)	98 (59.0%)	90 (40.7%)	193 (78.5%)
Indeterminate		196 (12.7%)	61 (12.8%)	54 (12.3%)	41 (12.6%)	40 (13.2%)		115 (12.3%)	44 (14.7%)	22 (13.3%)	28 (12.7%)	21 (8.5%)
Postural instability gait difficulty		606 (39.3%)	221 (46.4%)	144 (32.8%)	192 (59.1%)	49 (16.2%)		308 (33.0%)	127 (42.3%)	46 (27.7%)	103 (46.6%)	32 (13.0%)
Hoehn and Yahr stage*												
0–1.5	<0.001	808 (51.4%)	259 (53.6%)	298 (66.2%)	110 (33.4%)	141 (45.5%)	<0.001	216 (23.0%)	76 (24.9%)	60 (35.9%)	21 (9.5%)	59 (24.0%)
2–2.5		685 (43.6%)	211 (43.7%)	147 (32.7%)	181 (55.0%)	146 (47.1%)		660 (70.2%)	208 (68.2%)	103 (61.7%)	178 (80.2%)	171 (69.5%)
3		79 (5.0%)	13 (2.7%)	5 (1.1%)	38 (11.6%)	23 (7.4%)		64 (6.8%)	21 (6.9%)	4 (2.4%)	23 (10.4%)	16 (6.5%)
Untreated*	<0.001	149 (9.3%)	33 (6.7%)	69 (15.0%)	12 (3.6%)	35 (11.2%)	0.001	119 (12.6%)	35 (11.4%)	28 (16.8%)	14 (6.3%)	42 (17.0%)
LEDD total†	<0.001	293 (205)	304 (195)	245 (202)	361 (204)	272 (203)	<0.001	282 (212)	292 (196)	242 (206)	345 (225)	241 (209)
LEDD total on medication†§	<0.001	324 (190)	327 (183)	289 (188)	375 (195)	309 (188)	<0.001	327 (194)	333 (173)	293 (191)	368 (213)	297 (193)
Levodopa challenge†												
Percentage change	0.002	32.1 (22.8)	30.6 (23.0)	36.3 (24.0)	31.9 (21.7)	28.8 (20.9)	0.06	23.6 (15.2)	22.1 (15.5)	29.4 (16.7)	23.4 (16.0)	22.5 (12.3)

*χ^2^ test.

†Analysis of variance.

‡Changed denominator where 80% or more of questions were answered.

§The LEDD restricted to those who are taking dopaminergic medication.

Cluster 1 is *fast motor progression*; cluster 2 is *mild motor and non-motor disease*; cluster 3 is *severe motor disease, poor psychological well-being*and *poor sleep*; cluster 4 is *slow motor progression*.

LEDD, levodopa equivalent daily dose; UPDRS, Unified Parkinson’s Disease Rating Scale.

Within the Tracking Parkinson’s cohort, the L-dopa challenge was completed by 1021 (77.8%) out of 1313 patients who have had their 24-month visit. In the Discovery cohort, only 273 (35.5%) out of 770 patients completed the 18-month L-dopa challenge indicating a lack of power and potential selection bias in this data set. The mean percentage decrease in UPDRS III comparing pre with post challenge was greater in Tracking Parkinson’s than in Discovery (32.1% vs 23.6%). The change was highest in the *mild motor and non-motor disease* (2) cluster and slightly lower than average in the *slow* *motor* *progression* (4) cluster within both cohorts. There was strong evidence of a difference in response to L-dopa across the clusters in Tracking Parkinson’s (p=0.002), but not so strong in the smaller sample and potentially biased Discovery cohort (p=0.06).

### Comparison of prognosis by clusters between Tracking Parkinson’s and Discovery

**Figure 3:**
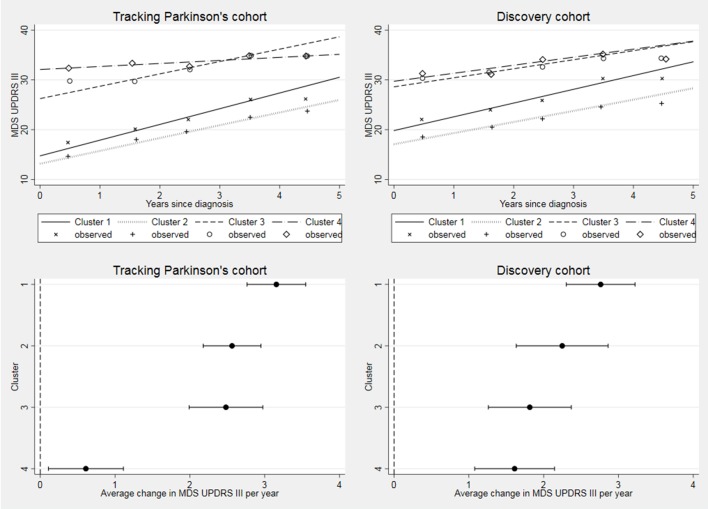
Longitudinal follow-up in MDS-UPDRS part III by cohort. Difference between clusters progression rates p<0.001 in Tracking Parkinson’s and p=0.007 in Discovery. Changed denominator where 80% or more of questions were answered. Observed data were split into yearly bins (0–1, 1–2, 2–3, 3–4 and 4–5 years) and the means plotted. MDS, Movement Disorders Society; UPDRS, Unified Parkinson’s Disease Rating Scale.

In Tracking Parkinson’s, 1421 (88.8%), 1154 (72.1%) and 204 (12.7%) have had 18-month, 36-month and 54-month assessment visits, respectively, with a median follow-up time of 3.0 years (IQR 1.8–3.2). In Discovery, 770 (81.6%), 490 (51.9%), 230 (24.4%) and 39 (4.1%) have had 18-month, 36-month, 54-month and 72-month assessment visits, respectively, with a median follow-up time of 3.0 years (IQR 1.5–4.4). All of the progression rates by cluster and cohort are shown in table 4. There was evidence of a significant difference in progression rates for the UPDRS III across clusters in Tracking Parkinson’s (p<0.001) and in Discovery (p=0.007). The same pattern of was seen in both cohorts. The *fast motor progression* (1) cluster had the fastest progression: 3.2 UPDRS III points per year in Tracking Parkinson’s and 2.8 points per year in Discovery, while the *slow motor progression* (4) cluster had the slowest motor progression, although the estimate for progression in the *slow motor progression*(4) cluster was markedly slower in Tracking Parkinson’s (0.6 UPDRS III points per year) than Discovery (1.6 points per year) and with hardly any overlap across the 95% CIs (see figure 3). Repeating the analysis using the UPDRS part II score (web supplementary figure 2), we found the same clusters in Tracking Parkinson’s with the fastest and slowest progression; however, in the Discovery cohort, we found no evidence of difference in progression rates.

10.1136/jnnp-2018-318337.supp3 Supplementary data

**Table 4:** Comparison of per-year progression rates within the two cohorts using the two approaches: multilevel random slope and intercept models (MLMs) versus pattern-mixture models (PMMs)

	Cluster	Tracking Parkinson's cohort		Discovery cohort	
		MLM slope estimate (95% CI)	PMM slope estimate (95% CI)	MLM slope estimate (95% CI)	PMM slope estimate (95% CI)
	1	3.16 (2.76 to 3.55)	3.08 (2.70 to 3.45)	2.76 (2.30 to 3.22)	2.66 (2.20 to 3.13)
MDS-UPDRS III	2	2.56 (2.18 to 2.95)	2.62 (2.23 to 3.01)	2.25 (1.63 to 2.86)	2.29 (1.72 to 2.87)
	3	2.48 (1.99 to 2.97)	2.66 (2.02 to 3.31)	1.81 (1.26 to 2.37)	1.79 (1.13 to 2.46)
	4	0.61 (0.11 to 1.11)	0.65 (0.09 to 1.21)	1.61 (1.08 to 2.15)	1.67 (1.04 to 2.30)
	P values	<0.001	<0.001	0.007	0.04
	1	−0.16 (−0.26 to −0.06)	−0.20 (−0.32 to −0.09)	−0.19 (−0.30 to −0.07)	−0.21 (−0.33 to −0.09)
MoCA adjusted	2	−0.02 (−0.12 to 0.08)	−0.04 (−0.12 to 0.04)	−0.10 (−0.25 to 0.05)	−0.09 (−0.24 to 0.05)
	3	−0.22 (−0.34 to −0.09)	−0.31 (−0.50 to −0.13)	−0.27 (−0.41 to −0.14)	−0.34 (−0.53 to −0.14)
	4	−0.04 (−0.17 to 0.08)	−0.10 (−0.29 to 0.08)	−0.17 (−0.30 to −0.04)	−0.20 (−0.34 to −0.06)
	P values	0.04	0.017	0.41	0.26
	1	1.63 (1.46 to 1.81)	1.61 (1.44 to 1.78)	1.43 (1.22 to 1.64)	1.44 (1.21 to 1.67)
MDS-UPDRS II	2	1.25 (1.08 to 1.42)	1.32 (1.13 to 1.51)	1.01 (0.73 to 1.28)	0.94 (0.68 to 1.19)
	3	1.51 (1.29 to 1.74)	1.68 (1.33 to 2.02)	1.25 (1.01 to 1.49)	1.41 (1.08 to 1.74)
	4	1.14 (0.92 to 1.37)	1.32 (1.02 to 1.62)	1.25 (0.99 to 1.51)	1.34 (1.04 to 1.63)
	P values	0.001	0.06	0.13	0.02

Cluster 1 is *fast motor progression*; cluster 2 is *mild motor and non-motor disease*; cluster 3 is *severe motor disease, poor psychological well-being*and *poor sleep*; cluster 4 is *slow motor progression*.

MDS, Movement Disorders Society; MoCA, Montreal Cognitive Assessment; UPDRS, Unified Parkinson’s Disease Rating Scale.

**Figure 4:**
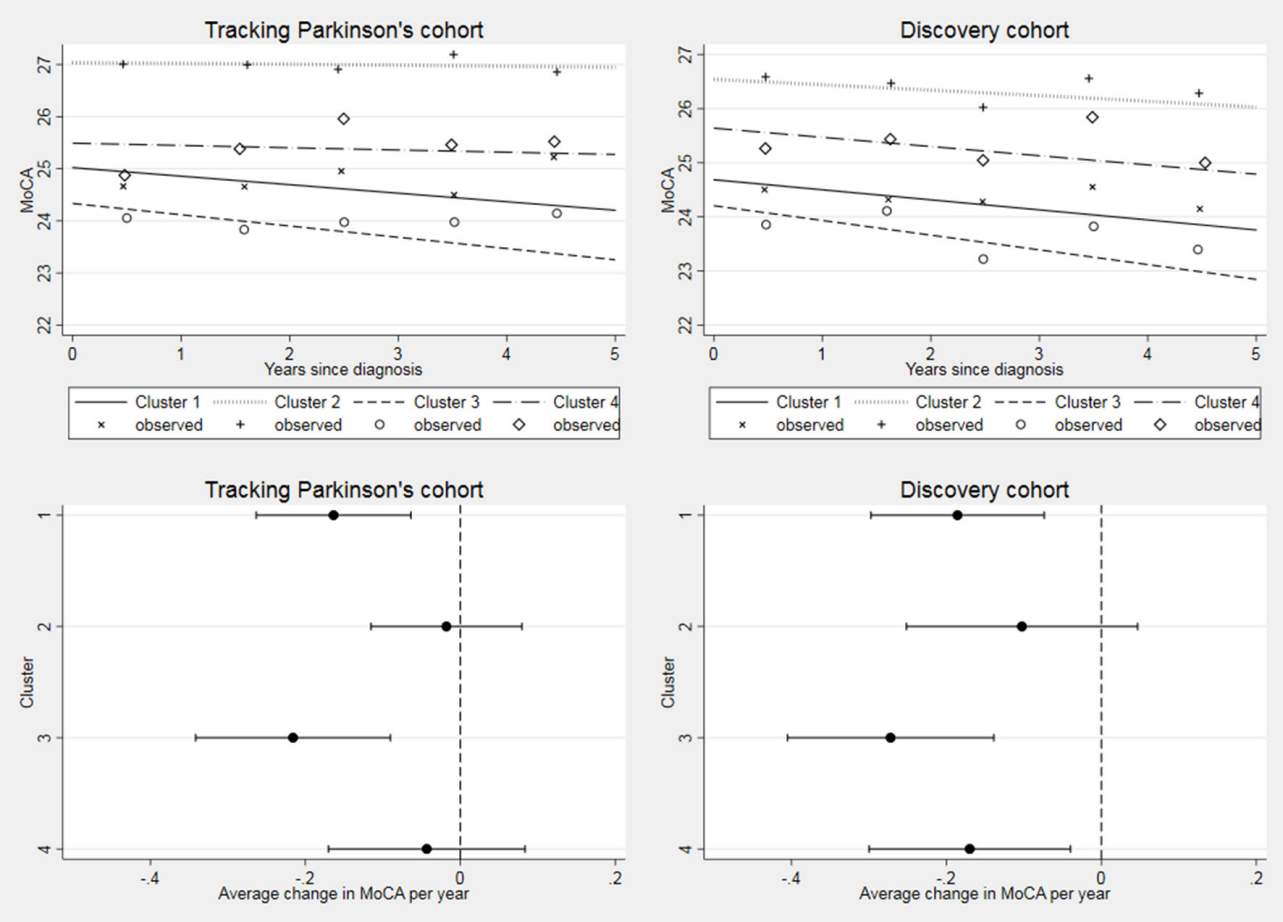
Longitudinal follow-up in Montreal Cognitive Assessment (MoCA) by cohort. Difference between cluster progression rates p=0.04 in Tracking Parkinson’s and p=0.41 in Discovery. Changed denominator where 80% or more of questions were answered. Observed data were split into yearly bins (0–1, 1–2, 2–3, 3–4 and 4–5 years) and the means plotted.

Cognitive decline, as measured by the MoCA, was fastest in the *severe motor disease, poor psychological well-being*and *poor sleep*(3) cluster in both cohorts (figure 4), but overall there was no significant difference in cognitive progression rates across clusters (Tracking Parkinson’s p=0.04; Discovery p=0.41).

Repeating our analyses using pattern-mixture models showed little difference in progression rate estimates (table 4), providing evidence that withdrawal has not biased our estimates. Adjusting the slope and intercept for baseline LEDD in our UPDRS III models, an attempt to see the effect that treatment had on progression rates, we found very similar rates (results not included).

No significant differences in motor UPDRS III progression were found between conventional tremor, PIGD and mixed clusters (web supplementary figure 3), although in Tracking (p<0.001), there was some evidence to suggest that those in the PIGD cluster have faster cognitive decline (web supplementary figure 4).

10.1136/jnnp-2018-318337.supp4 Supplementary data

10.1136/jnnp-2018-318337.supp5 Supplementary data

## Discussion

Our analyses identified four phenotypic subgroups among patients recently diagnosed with PD: (1) *fast motor progression* with symmetrical motor disease, poor olfaction, cognition and postural hypotension; (2) *﻿mild motor and non-motor disease* with intermediate motor progression; (3) *severe motor disease* (prominent bradykinesia/postural impairment), *poor psychological well-being* (mood, apathy, pain, fatigue) and *poor sleep* with intermediate motor progression; (4) *slow motor progression* with tremor-dominant, unilateral disease. The kappa statistic showed that the clusters calculated within the Discovery cohort were relatively stable compared with those predicted using the Tracking Parkinson’s cohort model even though some baseline characteristics differed significantly between the cohorts.

Our analysis has taken into account the five points recommended for studies using cluster analysis.^6^ (1) Our sample of patients with PD were all recently diagnosed and hence had more similar disease duration than other cross-sectional studies. (2) We used two sample populations of patients who have been well phenotyped across a wide a range of important domains. (3) We have taken into account the limitations of k-means by (a) using hierarchical clustering prior to the analysis to determine the number of clusters, (b) standardising all the variables so they have equal weighting and (c) using 500 random starts to prevent the selection of local optima. (4) We have looked at independent associations between our clusters with clinically meaningful variables such as response to L-dopa challenge and disease progression. (5) We have validated our approach using a second cohort collected using a nearly identical methodology.

Our previous paper reported five clusters in the Discovery cohort. The clusters in our new analysis are qualitatively similar although two of the original clusters (a) poor psychological well-being, rapid eye movement sleep behaviour disorder and sleep, and (b) severe motor and non-motor disease with poor psychological well-being have now merged into a single cluster (cluster 3). Our clusters are consistent with other similar studies in PD, which generally find a group with milder symptoms and a younger age at onset^3 5 15–22^ (our second cluster). Three studies also found a tremor-dominant group^17 18 20^ (our fourth cluster) and most studies find a group with more severe symptoms or rapid disease progression^3 4 15–22^ (our first and third clusters). Importantly, we have now demonstrated different rates of motor progression across our baseline-defined clusters, with a mean annual deterioration in UPDRS III scores varying significantly from 0.6 to 3.2 points (in Tracking Parkinson’s) between those with slowest and fastest progression. Interestingly, we also found, in keeping with another study^3^, that poor cognition and postural hypotension predicted faster motor progression.

### What is the clinical relevance of these findings?

Stratification, or defining different subcategories, is key to better understanding disease mechanisms and kinetics in PD, predicting disease course and ultimately delivering personalised management strategies. The emerging focus of PD trial design is on early motor disease, including novel immunomodulatory therapies that require intensive and invasive monitoring. Traditionally, little account has been taken of disease heterogeneity in early PD when selecting patients for randomised, placebo-controlled studies. However, our results show that baseline phenotype is associated with variable rates of subsequent motor progression, although confounded by potential medication response effects. The mean difference in UPDRS motor scores between the fastest and slowest motor progression subtypes in Tracking Parkinson’s was 2.6 points, equivalent to the primary hierarchical endpoint of several studies, including the ADAGIO study.^23^ Recruitment without taking into account heterogeneity and potential sources of recruitment bias may lead to less efficient designs, though there are various trade-offs between the cost of selecting patient subgroups, the sample size required for demonstrating a reduction in disease progression and increasing the length of follow-up.

### Strengths and limitations

This study has used two of the largest PD incidence cohorts worldwide. In addition, the methods were designed collaboratively with similar variables being collected using almost identical inclusion criteria, though the source of recruitment differed. While this may impact on the frequency of the subtypes of PD, it should not influence the consistency of the clusters or the within-cluster progression rates. It is possible that some patients will turn out to have other parkinsonian disorders, such as multiple system atrophy, despite only including those with a diagnostic probability of ≥90% at the latest visit, especially in the fast progression cluster. We had little missing data and we used imputation methods to reduce any bias. The association we found with levodopa response (which was analysed as relative change) may simply reflect the fact that the second cluster has milder disease, and since our estimates of motor function is carried out in the ‘on’ state, we would expect those with mild motor disease to be those who respond well to the medication. We are also limited by the proportion who completed the L-dopa challenge although the vast majority of those missing this data in the Tracking Parkinson’s cohort is due to them either not taking levodopa as part of their normal medication regime or not reaching the 24-month time point. Levodopa response is also composed of both short-duration and long-duration responses.^24^ Our levodopa challenge only measures the short-term response and our pre-dose scores are largely determined by the long-duration response. Also, the long-duration response is typically much larger than the short-duration response.

We used non-statistical criteria to help judge the best number of clusters, as the optimal number of clusters can differ depending on which statistic is the primary focus. Each cohort has its strengths and weaknesses. Tracking Parkinson’s is larger with more centres from a wider area of the UK population. The Discovery cohort used fewer clinicians to assess participants and had lower inter-rater variability. Discovery had more disabling disease and slightly worse cognitive function at baseline. Each cohort may have a slightly different mix of patients, but this will also occur in patients recruited for different clinical trials.

The major limitation in this analysis is that most of our data are restricted to the first 3 years of follow-up due to the studies being ongoing and patients not yet reaching 4.5 years of follow-up. We suspect this has reduced our power to detect differences between the clusters. The associations we saw between clusters and progression rates could be due to non-linearity of growth rates; however, non-linearity cannot be tested until the vast majority of patients have four or more observations.

We took a pragmatic perspective where disease progression estimates reflected both pathophysiology and treatment effects. An alternative approach is measurement of the untreated (underlying) progression of subtypes, which reduces potential confounding effects of dopaminergic therapy in modifying disease progression, and has been applied elsewhere.^25 26^ Accordingly the generalisability of our method may be limited if different treatment regimes were used in other clinical settings.

Neuropathological characterisation of the patient clusters at post mortem would help to address the question of the distribution and loads of α-synuclein, tau, vascular and amyloid pathology in driving both baseline clinical phenotype and subsequent motor and cognitive progression throughout the disease evolution of PD.^27^ It is intriguing to speculate whether patients in cluster 1, who have the fastest motor progression, prominent baseline non-motor symptoms, more symmetrical disease and a poor levodopa response, are defined by prominent cerebrovascular or amyloid pathologies. Clear delineation of patient subtypes is likely to introduce other potential therapeutic targets and lifestyle interventions to the clinical trials arena that look beyond pure α-synuclein-driven pathology. To date, a total of 345 subjects with PD (195 Tracking Parkinson’s, 150 Discovery cohort) have signed up to the nationally funded Parkinson’s UK Brain Donation programme, with six brains now available for neuropathological characterisation to begin to address these issues.

## Conclusion

We have found four clusters that replicate across two large independent cohorts of newly diagnosed patients with PD and which are associated with different responses to levodopa and motor progression rates. Future work should examine the reasons for these differences, and with longer follow-up and using growth mixture models, we should be able to identify more easily patient groups with different progression rates and how this relates to their baseline characteristics. This will also allow us to determine the robustness and clinical use of stratifying patients early in the disease course with better defined endpoints.
