# Supplementary material for: Developing and validating Parkinson’s disease subtypes and their motor and cognitive progression
Source: J Neurol Neurosurg Psychiatry. 2018 Jul 25;89(12):1279–87. doi: 10.1136/jnnp-2018-318337 (PMC6288789; doi:10.1136/jnnp-2018-318337)
Supplement: Supplementary data [file jnnp-2018-318337supp001.pdf]

E-appendix

Developing and validating Parkinson's Disease Subtypes and their motor and cognitive progression

E-appendix Methods

Patient evaluation

The Tracking Parkinson's cohort began measuring olfaction using the University of Pennsylvania Smell Identification Test (UPSIT) before changing to the Sniffin' sticks 16 item odour identification test when there became a difficulty in obtaining the UPSIT kits. The Discovery cohort only measured olfaction using the Sniffin' sticks 16 item odour identification test. We used IRT methods to convert the UPSIT scores to equivalent Sniffin' 16 scores[1]. We also used equipercentile methods to convert Leeds Anxiety and Depression scale into the more commonly used Hospital Anxiety and Depression scale.

We consider the L-dopa challenge as a percentage change by dividing the difference in pre and post dose total MDS-UPDRS III score measurements by the pre dose measure. A pragmatic levodopa challenge test was performed only in consenting patients who were already taking levodopa medication by the time of their 18 month (Discovery) or 24 month (Tracking Parkinson's) visit. Patients were asked to omit their usual levodopa dose approximately 12 hours before the morning challenge test. Patients who were also taking levodopa agonist medication, MAO-B or COMT inhibitors were also asked to omit these 12 hours before the challenge test (or 24 hours before if taking once daily dopamine agonist formulations). During the levodopa challenge, the patient was given their usual dose of oral levodopa with peripheral dopa-decarboxylase inhibitor, rather than a supramaximal standard dose of levodopa, and the MDS-UPDRS III performed at baseline and 1 hour later to assess response by a trained neurologist.

Statistical Analysis

Since k-means cluster analysis is not a statistical model per-say (it does not measure the uncertainty in any model estimates as it is just an algorithm) so it is not possible to use Rubin's rules to collate the 10 imputed datasets into one model. So for simplicity we used the data from our 10 multiply imputed datasets to create one single dataset (after carrying out the confirmatory factor analysis which is a statistical model) by taking the average for each variable across all 10 datasets. Also note that the amount of missing data we had was small and unlikely to bias our results in anyway. After taking into account those individuals who answered between 80-100% of a questionnaire in Tracking Parkinson's we had between 0.9%-5.3% missing baseline data (although the BFI and Sniffin' scores had ~10% missing data because they were collected at six months post baseline and were hence affected by drop-out) whilst in Discovery we had between 0.4% - 4.8% missing data.

Our factor analysis consisted of first an exploratory factor analysis in the Discovery cohort followed by a confirmatory factor analysis (CFA) in the Tracking Parkinson's cohort. In the CFA we examined the following goodness of fit statistics: Comparative Fit Index (CFI), Tucker-Lewis Index (TLI) and the Root Mean Square Error of Approximation (RMSEA). A model was considered to fit the data well if CFI was ≥ 0.90, TLI ≥ 0.90 and RMSEA ≤ 0.08 The same algorithm that produced the factor scores in Tracking Parkinson's was used in Discovery to ensure comparability between these variables across the cohorts.

Pattern-mixture models were estimated using a Structural Equation Modelling approach within Mplus. We constrained all the variances of the outcomes equal across the clusters and

time-points. The variances and covariance of the latent (random) intercept and slope were constrained to be equal across the clusters. Our pattern-mixture model was defined such that all patients withdrawing were considered the same, i.e withdrawing after visit 2 was considered to have the same effect on the intercept and slope as withdrawing after baseline. The numbers withdrawn in Tracking Parkinson's was 302/1601 (18.9%) and within Discovery 233/944 (24.7%).

E-appendix Results

Exploratory factor analysis (EFA)

In Discovery using the eigenvalue criteria we found 2 factors in each of the ten imputed datasets. The two factors identified were identical to the psychological well-being and non-tremor motor factors from the original paper EFA [2] (ignoring unavailable variables not in both datasets, that is the Purdue tests, the Get Up and Go Test and the flamingo test) except that the non-tremor motor factor also included the MoCA and semantic fluency variables.

Confirmatory factor analysis (CFA)

The CFA in the Tracking Parkinson's cohort using the variables from the EFA only met one of our pre-defined goodness of fit criteria with a CFI of 0.83, TLI of 0.87, and a RMSEA of 0.078. Although the cognitive and motor variables in the second factor might be highly correlated we thought that clinically it did not make sense to include them within the same factor. Dropping the two cognitive variables from this factor improved the goodness of fit with a CFI of 0.91, TLI of 0.93, and a RMSEA of 0.063. Therefore we excluded the cognitive variables from this factor with main manuscript Table 2 displaying the results from the resulting CFA. We named factor 1 "psychological well-being" and factor 2 "non-tremor motor" matching our original paper. The factor loadings varied from 0.31 – 0.86 and 0.42 – 0.77 in the two factors respectively. At this stage we excluded the other four BFI variables not loading into a factor and the UPDRS constipation variable for the sake of parsimony, which is the same approach used in our original paper. We also excluded the semantic fluency variable since we thought that the MoCA was a better measure of global cognitive function.

Cluster Analysis – choosing number of clusters

Web table 1 shows the statistics we used to determine the optimum number of clusters which pointed to the two and five cluster solutions in both Tracking Parkinson's and Discovery. So we used criteria other than these fit statistics to decide which was the optimum number of clusters.

We considered, initially, the agreement between our k-means clusters in the Discovery cohort and the clusters predicted from our Tracking Parkinson's discriminant model. The two cluster solution had excellent overall agreement (91.5%) and a kappa statistic consistent with "almost perfect" agreement (0.83) however because this only stratified individuals into a good and bad group this was not regarded as clinically that informative. The five cluster solution had the same overall agreement (67.9%) as the four cluster solution and a higher kappa statistic 0.60 compared to 0.58. However these kappa statistics are almost equivalent and both close to the borders of what would be considered "moderate" to "substantial" agreement. We chose the four cluster solution because it is more parsimonious than the five cluster.

Comparison of prognosis by clusters between Tracking Parkinson's and Discovery

We looked at what would happen if we relaxed the assumptions in our pattern-mixture model such that variances of the outcomes are equal across time-points or clusters and the variances/covariances of the random effects are equal across clusters. So we also fitted the

following models for UPDRS III and compared commonly used goodness of fit statistics like AIC, BIC as well as likelihood ratio tests.

1. Variances and covariance of random effects are different for the four clusters
2. Variances of the outcomes are different at each time-point
3. Combining assumptions for models 1 and 2 above.
4. As model 3 but variances of the outcomes are also different within each cluster

Using the goodness of fit statistics in Tracking PD we would select model 3 over our standard PMM model. However within all models the largest difference in mean progression rate (compared to standard PMM model) in any cluster was 0.22 UPDRS III points per year so almost identical to our default model. Using the same model (which was also favoured by AIC and likelihood ratio tests) in Discovery the largest difference in mean progression rate in any cluster was 0.12 UPDRS III points per year and the difference between clusters p-value increased from 0.04 to 0.10. However if we used the BIC to select a model in Discovery we would have selected the default model. Hence we are confident that the assumptions we made in our model has not made any impact on our progression rate estimates. We will further explore these issues along with non-linearity in a future paper when we have more longitudinal data available.

Web Table 1. Statistics to determine the number of clusters from the Ward hierarchical clustering. A higher value of Calinski/Harabasz pseudo-F index indicates more distinct clustering and a smaller value of the Duda/Hart pseudo-T squared indicates more distinct clustering. Bold indicates most distinct cluster.

	Tracking Parkinson's cohort		Discovery cohort	
Number of clusters	Calinski/Harabasz pseudo-F	Duda/Hart pseudo T-squared	Calinski/Harabasz pseudo-F	Duda/Hart pseudo T-squared
2	**163.4**	75.3	**96.2**	32.3
3	116.1	73.2	69.6	39.6
4	99.8	42.4	58.9	26.0
5	92.4	**37.2**	53.8	**18.1**

Web Table 2. Scores from each test within the four k-means clusters from both cohorts at baseline using the imputed data. Where standard cut-points exist in literature categorised scores are given as well as their total test scores. For standalone questions from the MDS-UPDRS scales the questions were dichotomised at 1 or above.

	Tracking Parkinson's clusters					Discovery clusters				
**Variable**	**Total N=1601**	**Cluster 1 N=493**	**Cluster 2 N=459**	**Cluster 3 N=336**	**Cluster 4 N=313**	**Total N=944**	**Cluster 1 N=218**	**Cluster 2 N=319**	**Cluster 3 N=196**	**Cluster 4 N=211**
UPDRS speech[a]	782 (48.8%)	251 (50.9%)	136 (29.6%)	233 (69.3%)	162 (51.8%)	445 (47.1%)	126 (57.8%)	95 (29.8%)	109 (55.6%)	115 (54.5%)
UPDRS rigidity	3.7 (2.9)	3.1 (2.7)	2.6 (2.2)	4.8 (3.2)	5.1 (2.9)	5.3 (2.7)	6.0 (2.9)	4.4 (2.3)	6.2 (2.8)	5.2 (2.5)
UPDRS bradykinesia	10.8 (7.0)	8.9 (5.8)	7.0 (4.9)	15.0 (7.3)	15.0 (6.5)	13.0 (6.5)	15.0 (6.1)	9.9 (5.2)	16.3 (6.9)	12.6 (5.9)
UPDRS Postural	2.6 (2.3)	2.2 (1.8)	1.6 (1.6)	3.8 (2.7)	3.2 (2.4)	2.6 (2.2)	3.1 (2.1)	1.7 (1.6)	3.8 (2.8)	2.1 (1.7)
UPDRS tremor	4.6 (3.8)	3.1 (2.9)	3.5 (2.7)	4.5 (3.5)	8.9 (3.8)	5.0 (3.8)	3.8 (3.4)	3.1 (2.5)	5.9 (3.8)	8.3 (3.2)
Percentage UPRDS III due to tremor	21.8 (17.6)	18.7 (18.7)	24.7 (20.2)	16.3 (12.9)	28.4 (12.0)	19.4 (14.2)	13.1 (11.5)	16.9 (14.8)	18.9 (11.7)	30.3 (11.8)
Laterality	6.4 (4.4)	4.3 (2.8)	5.5 (3.2)	6.3 (4.1)	11.0 (5.0)	7.1 (4.3)	4.0 (2.8)	6.7 (3.4)	7.4 (4.2)	10.5 (4.3)
Laterality (dichotomised unilateral[b])	1154 (72.1%)	290 (58.8%)	325 (70.8%)	245 (72.9%)	294 (93.9%)	725 (76.8%)	107 (49.1%)	257 (80.6%)	160 (81.6%)	201 (95.3%)
UPDRS apathy[a]	499 (31.2%)	123 (24.9%)	98 (21.4%)	184 (54.8%)	94 (30.0%)	194 (20.6%)	32 (14.7%)	43 (13.5%)	91 (46.4%)	28 (13.3%)
UPDRS fatigue[a]	1242 (77.6%)	365 (74.0%)	327 (71.2%)	307 (91.4%)	243 (77.6%)	670 (71.0%)	155 (71.1%)	220 (69.0%)	184 (93.9%)	111 (52.6%)
UPDRS pain[a]	894 (55.8%)	233 (47.3%)	220 (47.9%)	270 (80.4%)	171 (54.6%)	753 (79.8%)	173 (79.4%)	247 (77.4%)	179 (91.3%)	154 (73.0%)
BFI neuroticism	23.3 (6.4)	22.1 (5.8)	22.1 (6.5)	26.4 (6.3)	23.7 (6.3)	22.3 (6.5)	20.9 (6.6)	21.6 (5.9)	26.4 (6.0)	20.9 (6.3)
HADS anxiety	5.3 (4.2)	3.9 (3.0)	4.0 (3.5)	9.3 (4.3)	5.1 (3.8)	4.6 (3.8)	3.4 (2.8)	4.1 (3.1)	8.2 (4.2)	3.1 (2.8)
HADS anxiety (dichotomised[c])	442 (27.6%)	66 (13.4%)	76 (16.6%)	215 (64.0%)	85 (27.2%)	197 (20.9%)	21 (9.6%)	50 (15.7%)	109 (55.6%)	17 (8.1%)
HADS depression	4.6 (3.4)	3.6 (2.6)	3.2 (2.6)	8.2 (3.1)	4.4 (2.9)	4.4 (3.4)	3.7 (2.6)	3.8 (2.8)	8.0 (3.4)	2.8 (2.5)

HADS depression (dichotomised[c])	310 (19.4%)	37 (7.5%)	29 (6.3%)	194 (57.7%)	50 (16.0%)	168 (17.8%)	15 (6.9%)	34 (10.7%)	102 (52.0%)	17 (8.1%)
QUIP	350 (21.9%)	89 (18.1%)	87 (19.0%)	113 (33.6%)	61 (19.5%)	204 (21.6%)	30 (13.8%)	71 (22.3%)	74 (37.8%)	29 (13.7%)
RBD	4.0 (2.6)	3.8 (2.4)	3.0 (1.9)	6.6 (2.5)	3.2 (2.1)	4.1 (2.6)	4.5 (2.6)	3.4 (2.1)	6.1 (2.7)	2.7 (1.7)
RBD (dichotomised[d])	574 (35.9%)	162 (32.9%)	81 (17.6%)	256 (76.2%)	75 (24.0%)	325 (34.4%)	94 (43.1%)	76 (23.8%)	127 (64.8%)	28 (13.3%)
ESS	6.8 (4.5)	6.0 (3.5)	4.8 (3.3)	11.1 (4.8)	6.0 (3.8)	7.6 (4.5)	7.0 (4.0)	6.5 (3.9)	11.3 (4.5)	6.3 (3.6)
ESS (dichotomised[e])	295 (18.4%)	48 (9.7%)	28 (6.1%)	180 (53.6%)	39 (12.5%)	230 (24.4%)	41 (18.8%)	51 (16.0%)	113 (57.7%)	25 (11.8%)
MoCA	25.4 (3.4)	24.7 (3.3)	27.1 (2.3)	24.0 (4.0)	25.4 (3.1)	25.0 (3.3)	23.3 (3.3)	26.5 (2.4)	24.0 (3.5)	25.4 (3.2)
MoCA[f]- Normal	1211 (75.6%)	339 (68.8%)	428 (93.2%)	208 (61.9%)	236 (75.4%)	671 (71.1%)	113 (51.8%)	283 (88.7%)	112 (57.1%)	163 (77.3%)
MoCA[f]- MCI	183 (11.4%)	77 (15.6%)	18 (3.9%)	46 (13.7%)	42 (13.4%)	145 (15.4%)	45 (20.6%)	27 (8.5%)	47 (24.0%)	26 (12.3%)
MoCA[f]- Demented	207 (12.9%)	77 (15.6%)	13 (2.8%)	82 (24.4%)	35 (11.2%)	128 (13.6%)	60 (27.5%)	9 (2.8%)	37 (18.9%)	22 (10.4%)
Sniffin'	7.7 (2.9)	5.7 (1.9)	10.2 (2.2)	7.2 (2.9)	7.6 (2.5)	7.1 (2.9)	5.3 (2.1)	8.5 (2.7)	7.2 (2.8)	6.9 (2.7)
Sniffin' – hyposmic[g]	1180 (73.7%)	439 (89.0%)	251 (54.7%)	256 (76.2%)	234 (74.8%)	743 (78.7%)	193 (88.5%)	232 (72.7%)	143 (73.0%)	175 (82.9%)
UPDRS hallucinations[a]	141 (8.8%)	31 (6.3%)	11 (2.4%)	83 (24.7%)	16 (5.1%)	105 (11.1%)	32 (14.7%)	23 (7.2%)	44 (22.4%)	6 (2.8%)
Systolic postural drop	4.2 (13.6)	7.8 (13.1)	-0.4 (11.8)	8.3 (14.7)	1.2 (13.1)	6.3 (15.9)	19.6 (16.8)	-0.7 (12.1)	6.1 (14.5)	3.2 (12.4)
Constipation[h]	529 (33.0%)	176 (35.7%)	115 (25.1%)	144 (42.9%)	94 (30.0%)	459 (48.6%)	153 (70.2%)	117 (36.7%)	103 (52.6%)	86 (40.8%)
UPDRS urinary[a]	942 (58.8%)	262 (53.1%)	243 (52.9%)	261 (77.7%)	176 (56.2%)	594 (62.9%)	141 (64.7%)	168 (52.7%)	177 (90.3%)	108 (51.2%)

[a]Dichotomised individual UPDRS questions at 1 or more

[b]Dichotomised Laterality at a difference of four or more between left side and right side

[c]Dichotomised HADS at 8 or more

[d]Dichotomised RBD at 5 or more

[e]Dichotomised ESS at 11 or more

[f]Categorised MoCA at ≤ 21 = Dementia, 22-23 = MCI, or 24+ = Normal

[g]Dichotomised Sniffin' at or below the 15th centile by age and gender group
[h]Dichotomised Constipation <1 bowel movement per day or laxative use

Web Table 3. Agreement of patients in Discovery classified in each cluster: Kmeans on Discovery vs predicted clusters from Tracking Parkinson's discriminant analysis model

	Predicted 1	Predicted 2	Predicted 3	Predicted 4
Kmeans 1	160	0	44	14
Kmeans 2	110	154	22	33
Kmeans 3	6	3	157	30
Kmeans 4	31	10	0	170

Overall agreement 67.9%
Kappa (95% CI): 0.58 (0.54, 0.61)

WEB FIGURE LEGENDS

Web figure 1. Flow chart for entry into this analysis

Web Figure 2. Longitudinal follow up in MDS-UPDRS part II by cohort Difference between clusters progression rates p=0.001 in Tracking Parkinson's and p=0.13 in Discovery. Changed denominator where 80% or more of questions were answered Observed data was split into yearly bins (0-1,1-2,2-3,3-4 and 4-5 years) and the means plotted.

Web Figure 3. Longitudinal follow up in MDS-UPDRS part III by cohort looking at conventional clusters (TD, PIGD, mixed). Difference between clusters progression rate p=0.21 in Tracking Parkinson's and p=0.95 in Discovery. Changed denominator where 80% or more of questions were answered. Observed data was split into yearly bins (0-1,1-2,2-3,3-4 and 4-5 years) and the means plotted.

Web Figure 4. Longitudinal follow up in MoCA by cohort looking at conventional clusters (TD, PIGD, mixed). Difference between clusters progression rate p<0.001 in Tracking Parkinson's and p=0.33 in Discovery. Changed denominator where 80% or more of questions were answered. Observed data was split into yearly bins (0-1,1-2,2-3,3-4 and 4-5 years) and the means plotted.

eReferences

1. Lawton M, Hu MT, Baig F, et al. Equating scores of the University of Pennsylvania Smell Identification Test and Sniffin' Sticks test in patients with Parkinson's disease. *Parkinsonism Relat Disord*. 2016.
2. Lawton M, Baig F, Rolinski M, et al. Parkinson's Disease Subtypes in the Oxford Parkinson Disease Centre (OPDC) Discovery Cohort. *J Parkinsons Dis.* 2015;5(2):269-279.
