# Supplementary figures and images for: Developing and validating Parkinson’s disease subtypes and their motor and cognitive progression
Source: J Neurol Neurosurg Psychiatry. 2018 Jul 25;89(12):1279–87. doi: 10.1136/jnnp-2018-318337 (PMC6288789; doi:10.1136/jnnp-2018-318337)

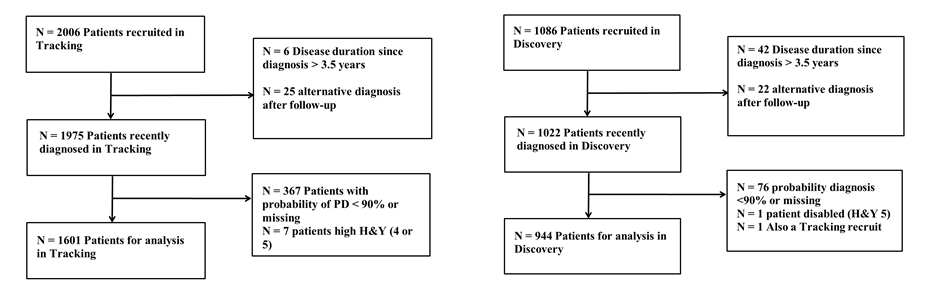

Supplement: Supplementary data [file jnnp-2018-318337supp002.tif]

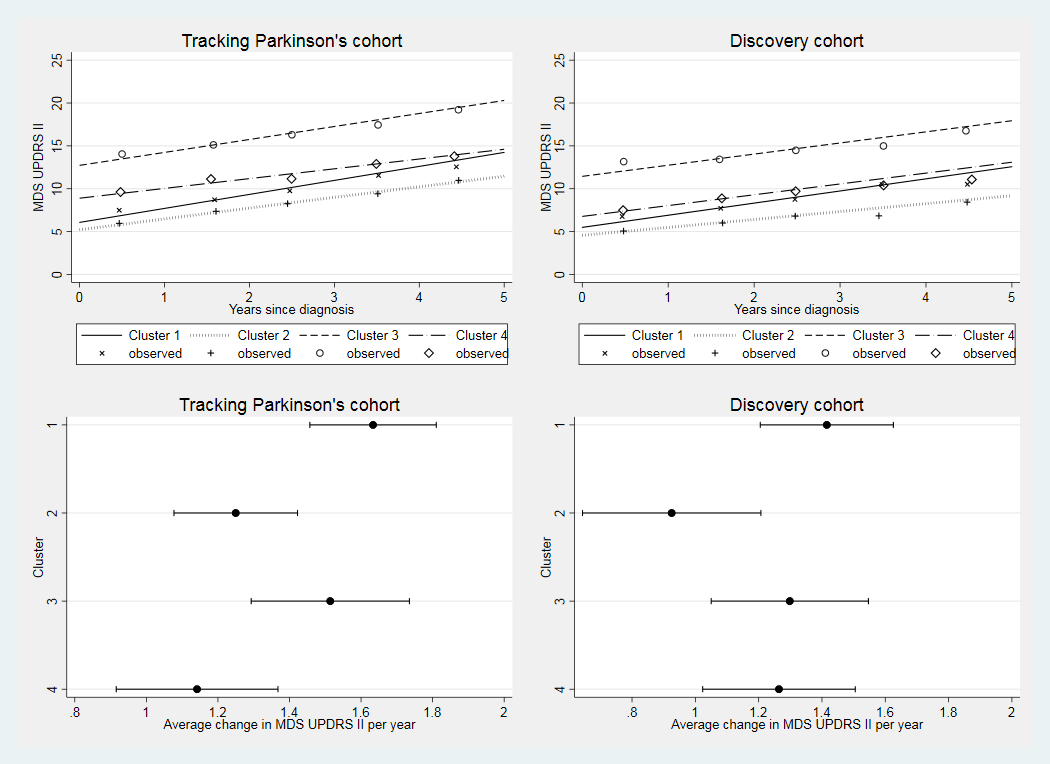

Supplement: Supplementary data [file jnnp-2018-318337supp003.tif]

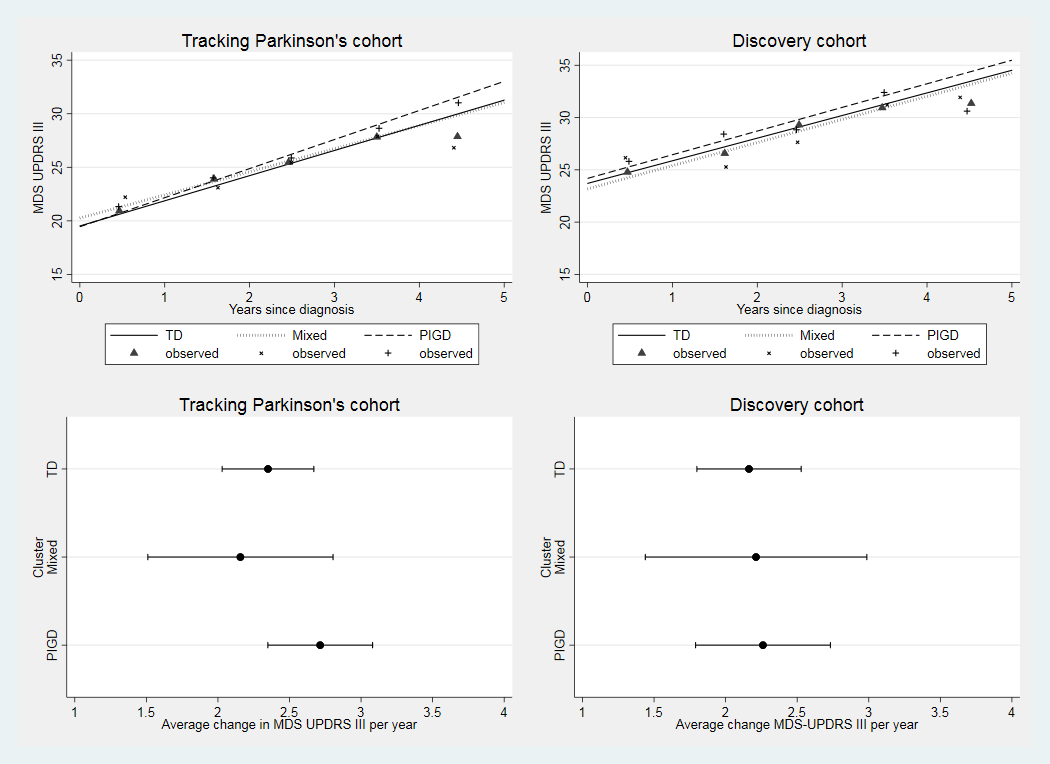

Supplement: Supplementary data [file jnnp-2018-318337supp004.tif]

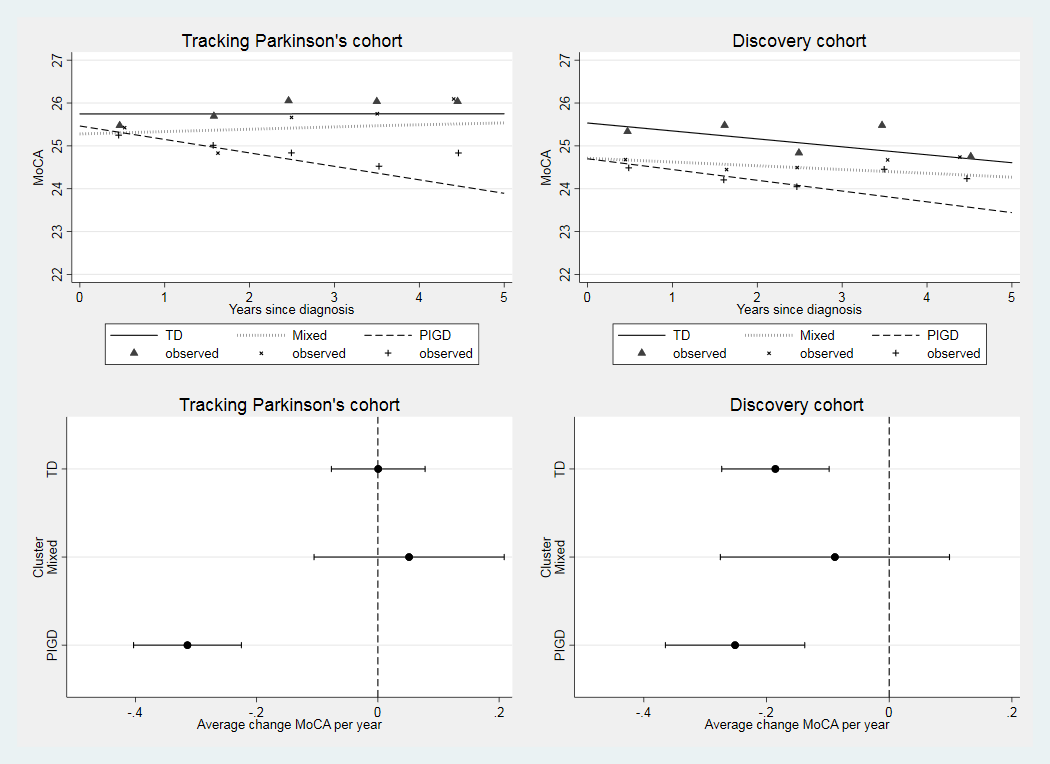

Supplement: Supplementary data [file jnnp-2018-318337supp005.tif]
